# Supplementary material for: Phytochemical Profile, Safety and Efficacy of a Herbal Mixture Used for Contraception by Traditional Health Practitioners in Ngaka Modiri Molema District Municipality, South Africa
Source: Plants (Basel). 2022 Jan 12;11(2):193. doi: 10.3390/plants11020193 (PMC8778889; doi:10.3390/plants11020193)
Supplement: Supplementary file 1 [file plants-11-00193-s001.zip › plants-1523420-supplementary.pdf]

Supplementary Materials

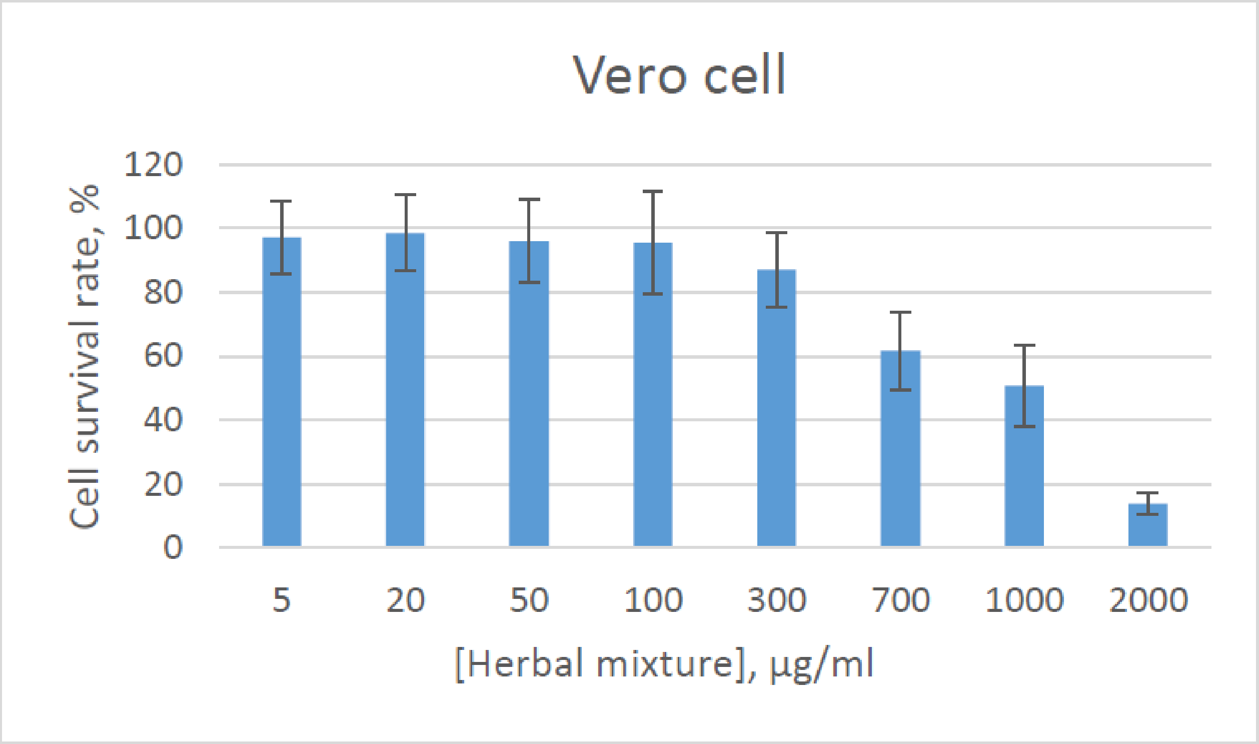

Figure S1: Cell survival (%) of Vero cells after 72 h treatment with herbal mixture based on 3-(4,5-dimethylthiazol-2-yl)-2,5-diphenyl tetrazolium bromide (MTT) assay. Values are mean, standard deviation, n =6
